# Efficacy of hypertonic dextrose injection (prolotherapy) in temporomandibular joint dysfunction: a systematic review and meta-analysis

**DOI:** 10.1038/s41598-021-94119-2

**Published:** 2021-07-19

**Authors:** Regina Wing-Shan Sit, Kenneth Dean Reeves, Claire Chenwen Zhong, Charlene Hoi Lam Wong, Bo Wang, Vincent Chi-ho Chung, Samuel Yeung-shan Wong, David Rabago

**Affiliations:** 1 The Jockey Club School of Public Health and Primary Care, Faculty of Medicine, The Chinese University of Hong Kong, Sha Tin, New Territories Hong Kong, China; 2 Private Practice, Roeland Park, KS USA; 3 Department of Family and Community Medicine, Pennsylvania State University, Pennsylvania, USA

**Keywords:** Pain management, Medical research

## Abstract

Hypertonic dextrose prolotherapy (DPT) has been reported to be effective for temporomandibular disorders (TMDs) in clinical trials but its overall efficacy is uncertain. To conduct a systematic review with meta-analysis of randomized controlled trials (RCTs) to synthesize evidence on the effectiveness of DPT for TMDs. Eleven electronic databases were searched from their inception to October, 2020. The primary outcome of interest was pain intensity. Secondary outcomes included maximum inter-incisal mouth opening (MIO) and disability score. Studies were graded by “Cochrane risk of bias 2” tool; if data could be pooled, a meta-analysis was performed. Ten RCTs (n = 336) with some to high risk of bias were included. In a meta-analysis of 5 RCTs, DPT was significantly superior to placebo injections in reducing TMJ pain at 12 weeks, with moderate effect size and low heterogeneity (Standardized Mean Difference: − 0.76; 95% CI − 1.19 to − 0.32, I^2^ = 0%). No statistically significant differences were detected for changes in MIO and functional scores. In this systematic review and meta-analysis, evidence from low to moderate quality studies show that DPT conferred a large positive effect which met criteria for clinical relevance in the treatment of TMJ pain, compared with placebo injections.

Protocol registration at PROSPERO: CRD42020214305.

## Introduction

Temporomandibular disorders (TMDs) are a group of conditions defined by anatomical, histological, and/or functional abnormalities of the muscular and/or articular components of temporomandibular joint (TMJ). They are characterized by pain located over the TMJ or surrounding tissues, and functional limitations of jaw movements such as chewing difficulty, jaw fatigue, grinding of teeth, tension about the jaw, or clicking with jaw motion^1^. The incidence of the first painful TMDs is 3–4% per annum and primarily affects young and middle aged adults with a prevalence of 5–10%^2,3^. While its natural history is not well studied, TMDs have been reported as recurrent in 65% and chronic in 19% of the affected population^2^. Treatment and research of TMD is complicated by the varied etiology and diagnostic criteria, which have been organized as the Research Diagnostic Criteria (RDC/TMD) and classified by likely etiology^4^. The extent to which RDC/TMD classification can inform guideline-driven care is not yet known.

International consensus regarding clinical management of TMDs has advocated the use of non-surgical therapeutic modalities for TMDs^5^. In addition to education and self-care techniques, the use of simple analgesics, occlusion splints, physiotherapy and acupuncture have been suggested; however, systematic reviews have not detected overall superiority of any one therapy^6–8^. Evidence on the use of injection therapies for TMDs is limited; options include intra-articular corticosteroids for inflammatory TMDs^9^, hyaluronic acid for TMJ osteoarthritis^10,11^, and intramuscular botulinum toxin for TMJ myofascial pain^12^. However, most of these studies were characterized by small sample size, short study period, lack of methodologic rigor and inconsistent results, which limit the ability to draw consistent recommendations in for clinical practice^5,13^.

Hypertonic dextrose prolotherapy (DPT) is an injection therapy used to treat chronic painful musculoskeletal conditions^14,15^. The mechanism of action is not well understood; the historical understanding posits that DPT facilitates healing and subsequent pain control through initiation of a temporary inflammatory reaction with related tissue proliferation^16–19^. Recent literature also suggests the mechanism is multifactorial and may include direct sensorineural effects^20^. Recently, a growing number of methodologically higher quality clinical trials have evaluated the use DPT for TMDs, which reported beneficial effects on pain and dysfunction using standardized outcomes^21,22^. However, the findings were not included in the previous systematic review^23^. Patients, clinicians and health care systems benefit from ongoing review of changing medical literature to assist clinical decision making informed by the best available evidence^24^.

The aim of this study was to conduct a systematic review of randomized control trials (RCTs) to assess and analyze the overall efficacy of DPT in TMDs. We hypothesized that DPT would reduce pain and improve function of TMJs, compared to placebo interventions, among patients with TMDs.

## Results

**Figure 1:**
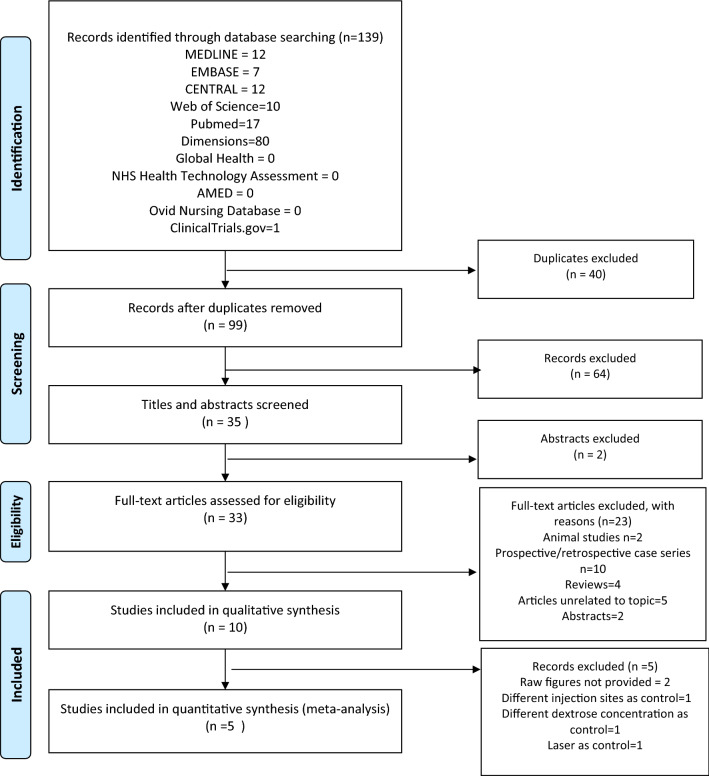
PRISMA 2009 flow diagram.

We identified 99 citations from all searches after excluding 40 duplicates. After screening the titles and abstracts, we retrieved 33 full texts for further assessment. Of these, 23 were excluded for the following reasons: duplicate publication as conference abstract (n = 2), trial without a control arm (n = 10), narrative review (n = 4), animal study (n = 2) and articles not related to the topic (n = 5). Ten full texts were included for descriptive synthesis^21,22,25–31^, among which 5 were included in quantitative synthesis^21,22,25,26,28^ (Fig. 1) .

### Characteristics of included trials

Characteristics of 10 included trials was summarized in Table 1. The sample sizes of the studies ranged from 12 to 72, with a total of 336 individuals. The study period ranged from 4 weeks to 1 year post-enrollment. The injection protocols consisted of intra-articular injection only, or a combined approach of intra and extra-articular injections. The injection frequency ranged from single injection to 4 injections, weekly to 4 weeks apart, with dextrose concentration varying from 10 to 30% (Table 1) .

**Table 1:** Study characteristics table.

	Title	Year	Sample size	Sample analyzed	Intervention group	Control group(s)	Mean age	Female (%)	DPT Inj. sites	Dextrose volume/inj	DPT inj. frequency	Outcomes	Assessment time points	Duration (weeks)
1	The Efficacy of dextrose prolotherapy for temporomandibular joint hypermobility: a preliminary prospective, randomized, double-blind, placebo-controlled clinical trial	Refai 2011	N = 12	N = 12	Gp A (n = 6):2 ml 10% dextrose + 1 ml 2% mepivacaine	Gp B (n = 6): 2 ml NS + 1 ml 2% mepivacaine	26.42 ± 5.66	83.30%	IA (superior joint space) Superior and inferior capsular attachment	3 ml	4 inj.; 6-week apart	*Pain (4 scales: no, mild, moderate and severe) Number of luxations (locking /month) MMO (cm)	Week 0, 6, 12, 18 and 30	30
2	Is dextrose prolotherapy superior to placebo for the treatment of temporomandibular joint hypermobility? A randomized clinical trial	Kilic 2016	N = 30	N = 26	Gp A (n = 14): 2 ml dextrose 30% dextrose + 2 ml NS + 1 ml 2% mepivacaine	Gp B (n = 12): 4 ml NS + 1 ml 2% mepivacaine	30.81 ± 11.60	73%	IA (superior joint space) posterior disc attachment Superior and inferior capsular attachment Stylo-mandibular ligament	5 ml	3 inj.; 4-week apart	Vas pain 0–10 Masticatory efficiency VAS 0–10 Joint sounds VAS 0–10 Painless mouth openning mm MMO (mm) Lateral motion (mm) Protrusion motion (mm)	0, 52	52
3	Change of site of intra-articular injection of hypertonic dextrose resulted in different effects of treatment	Fouda 2018	N = 72	N = 72	25% dextrose + 2% mepivaine Gp A (n = 18):sup. Joint space	25% dextrose + 2% mepivaine at different injection sites:Gp B (n = 18):capsule Gp C (n = 18): inferior joint space Gp D (n = 18): retrodiscal tisse	Mean 30 (SD 18–42)	77.80%	Gp A: superior jt space Gp B: capsule Gp C: inferior jt space Gp D : retrodiscal tissue	1.5 ml	4 inj.; weekly	VAS 0–100	Week 0, 2, 12	12
4	Evaluation of the efficacy of different concentrations of dextrose prolotherapy in temporomandibular joint hypermobility treatment	Mustafa 2018	N = 40	N = 37	Gp A (n = 9): 1.5 ml 20% dextrose + 1.5 ml 2% lidacaine	Gp B (n = 10) : 1.5 ml 10% dextrose + 1.5 ml 2% lidacaine Gp C (n = 9): 1.5 ml 30% dextrose + 1.5 ml 2%lidacaine Gp D (n = 9): 1.5 ml NS + 1.5 ml 2% lidocaine	25 ± 6.54	70%	IA (superior joint space) Posterior disc attachment Superior and inferior capsular attachment	3 ml	4 inj.; 4 weeks apart	VAS 0–10 MMO (mm) Luxation per month( yes/no) Joint sounds (yes/no)	week 0, 4, 8, 12, 16	16
5	Treatment of temporomandibular dysfunction with hypertonic dextrose injection (Prolotherapy): a randomized controlled trial with long-term partial crossover	Louw 2018	N = 42	N = 40	Gp A (n = 22): 20% dextrose + 0.2% lidocaine	Gp B (n = 20): water + 0.2% lidocaine	46 ± 14	83%	IA (superior joint space)	1 ml	3 inj.; 4 weeks apart	NRS 0–10 Pain NRS 0–10 function MIO (mm)	week 0, 4, 8, 12, 52	52 (open label after week 12)
6	Sodium hyaluronic acid, platelet rich plasma and dextrose prolotherapy in management of temporo-mandibular joint internal derangement. A comparative study	Mahmoud 2018	N = 45	not reported	Gp A (n = 15):12.5 dextrose + 2% lidocaine	Gp B (n = 15 ): hyaluronic acid Gp C (n = 15): platelet rich plasma	Age range (20–50)	62.20%	IA (posterior joint space) Anterior disc attachement Messeter muscle attachment	3 ml	3 inj.; 2 weeks apart	*VAS 0–10 *MIO (mm) *Mandibular deviation (yes/no)	Week 0, 4, 12, 24, 52	52
7	Dextrose prolotherapy in the treatment of recurrent temporomandibular joint dislocation (clinical study)	Saadat 2018	N = 16	N = 16	25% dextrose + 2% lidocaine Gp A (n = 8) : superior joint space	25% dextrose + 2% lidocaine Gp B (n = 8) : retrodiscal ligamament	29.5 (age range 23 to 40 )	69%	Gp A-superior joint space ; Gp B -retrodiscal ligament	2 ml	Single inj. at week 0	*VAS 0–10 *MIO (cm) *Number of dislocation per week	Week 0, 2, 4, 12, 24	24
8	Assessment of the therapeutic effects for autologous blood versys dextrose prolotherapy for the treatment of temporo-mandibular joint hypermobility: a randomized prospective clinical study	Arafat 2019	N = 30	Not reported	Gp A (n = 15):10% dextrose + 2% mepivacaine	Gp B (n = 15): autologous blood	18–39 years old	37%	IA (superior joint space) superior and inferior capsular attachment	3 ml	3 inj. 2-weeks apart	*VAS 0–10 *MIO (mm)	Week 0, 2, 12, 24	24
9	Dextrose prolotherapy versus lidocaine injection for temporomandibular dysfunction: a pragmatic randomized controlled tria^la^	Zarate 2020	N = 29	N = 27	GP A (n = 15): 20% dextrose + 0.2% lidocaine	Gp B (n = 14): water + 0.2% lidocaine	47 ± 17	86%	IA (superior joint space)	1 ml	3 inj.; 4-weeks apart	NRS 0–10 Pain NRS 0–10 function MIO (mm)	Week 0, 4, 8, 12, 52	52 (open label after week 12)
10	Dextrose prolotherapy versus low level laser therapy (LLLT) for Management of temporomandibular joint disorders (TMD): clinical randomized controlled study	Hassanien 2020	N = 20	N = 20	Gp A (n = 10): 12.5% dextrose + 2% lidocaine	Gp B (n = 10): laser (3 times per week for 4 weeks)	26 ± 4	50%	IA (posterior joint space) Anterior disc attachement Messeter muscle attachment	3 ml	3 inj.; 2-week apart	VAS 0–10 MMO (mm)	Week 2, 4	4

*Gp* group, *DPT* hypertonic dextrose prolotherapy, *IA* Intra-articular, *VAS* visual analog scale, *NRS* numerical rating scale, *MIO* maximum incisor opening, *MM* minimeter, *NS* normal saline.

*Raw figures not provided.

### Risk of bias assessment

In the domain of “bias arising from randomization process”, 2 studies had low bias^22,27^, and 8 had some bias^21,25,26,28–32^. In the domain of “bias due to deviations from intended interventions, 3 studies had high bias^26,28,29^, 2 had some bias^31,32^ and 5 had low bias^21,22,25,27,30^. In the domain of “bias due to missing outcome data”, 3 had some bias^26,28,29^, and 7 had low bias^21,22,25,27,30–32^. In the domain of “bias in measurement of outcome”, 7 had some bias^26–32^, and 3 with had low bias^21,22,25^. In the domain “bias in selection of reported outcome”, 1 had high bias^27^, 8 had some bias^22,25,26,28–32^ and 1 had low bias^21^. Overall, the risk of bias assessment amongst included studies was “some” to “high” (Table 2).

**Table 2:** Details of signaling questions in each domain of risk of bias assessment for 10 randomized controlled trials.

Domains	Signaling questions	Reponses of RCTs									
		Refai 2011	Kilic 2016	Fouda 2018	Mustafa 2018	Louw 2018	Mahmoud 2018	Saadat 2018	Arafat 2019	Zarate 2020	Hassanien 2020
Bias arising from the randomization process	1.1 Was the allocation sequence random?	NI	NI	PY	NI	Y	NI	NI	NI	Y	NI
	1.2 Was the allocation sequence concealed until participants were recruited and assigned to interventions?	NI	NI	Y	NI	Y	NI	NI	NI	Y	NI
	1.3 Did baseline differences between intervention groups suggest a problem with the randomization process?	NI	N	NI	N	PY	NI	NI	NI	PN	NI
	RoB judegement	SOME	SOME	LOW	SOME	SOME	SOME	SOME	SOME	LOW	SOME
Bias due to deviations from intended interventions	2.1 Were participants aware of their assigned intervention during the trial?	N	PN	PN	PN	PN	PY	PN	PY	N	PY
	2.2. Were carers and people delivering the interventions aware of participants’ assigned intervention during the trial?	N	PN	PN	PN	PN	PY	PN	PY	N	PY
	2.3. If Y/PY/NI to 2.1 or 2.2: Were there deviations from the intended intervention that arose because of the trial context?	NA	NA	NA	NA	NA	NI	NA	NI	NA	NI
	2.4 If Y/PY to 2.3: Were these deviations likely to have affected the outcome?	NA	NA	NA	NA	NA	NA	NA	NA	NA	NA
	2.5. If Y/PY/NI to 2.4: Were these deviations from intended intervention balanced between groups?	NA	NA	NA	NA	NA	NA	NA	NA	NA	NA
	2.6 Was an appropriate analysis used to estimate the effect of assignment to intervention?	PY	PN	PY	N	Y	NI	PY	PY	PY	PY
	2.7 If N/PN/NI to 2.6: Was there potential for a substantial impact (on the result) of the failure to analyse participants in the group to which they were randomized?	NA	PY	NA	PY	NA	NI	NA	NA	NA	NA
	RoB judegement	LOW	HIGH	LOW	HIGH	LOW	HIGH	LOW	SOME	LOW	SOME
Bias due to missing outcome data Bias in measurement of the outcome	3.1 Were data for this outcome available for all, or nearly all, participants randomized?	Y	N	PY	N	Y	NI	PY	PY	Y	PY
	3.2 If N/PN/NI to 3.1: Is there evidence that the result was not biased by missing outcome data?	NA	PN	NA	PN	NA	PN	NA	NA	NA	NA
	3.3 If N/PN to 3.2: Could missingness in the outcome depend on its true value?	NA	NI	NA	NI	NA	NI	NA	NA	NA	NA
	3.4 If Y/PY/NI to 3.3: Is it likely that missingness in the outcome depended on its true value?	NA	PN	NA	PN	NA	PN	NA	NA	NA	NA
	RoB judegement	LOW	SOME	LOW	SOME	LOW	SOME	LOW	LOW	LOW	LOW
	4.1 Was the method of measuring the outcome inappropriate?	PN	PN	PN	PN	PN	PN	PN	PN	PN	PN
	4.2 Could measurement or ascertainment of the outcome have differed between intervention groups?	PN	PN	PN	PN	PN	PN	PN	PN	PN	PN
	4.3 If N/PN/NI to 4.1 and 4.2: Were outcome assessors aware of the intervention received by study participants?	PN	NI	NI	NI	PN	NI	NI	NI	N	NI
	4.4 If Y/PY/NI to 4.3: Could assessment of the outcome have been influenced by knowledge of intervention received?	NA	PY	PY	PY	NA	PY	PY	PY	NA	PY
	4.5 If Y/PY/NI to 4.4: Is it likely that assessment of the outcome was influenced by knowledge of intervention received?	NA	PN	PN	PN	NA	PN	PN	PN	NA	PN
	RoB judegement	LOW	SOME	SOME	SOME	LOW	SOME	SOME	SOME	LOW	SOME
Bias in selection of the reported result	5.1 Were the data that produced this result analysed in accordance with a pre-specified analysis plan that was finalized before unblinded outcome data were available for analysis?	NI	NI	NI	NI	PY	NI	NI	NI	NI	NI
	For 5.2 and 5.3 Is the numerical result being assessed likely to have been selected, on the basis of the results, from…										
	5.2. … multiple eligible outcome measurements (e.g. scales, definitions, time points) within the outcome domain?	PN	PN	PY	PN	PN	PN	PN	PN	PN	PN
	5.3 … multiple eligible analyses of the data?	PN	PN	PN	PN	PN	PN	PN	PN	PN	PN
	RoB judegement	SOME	SOME	HIGH	SOME	LOW	SOME	SOME	SOME	SOME	SOME
Overall bias		SOME	HIGH	HIGH	HIGH	SOME	HIGH	SOME	SOME	SOME	SOME

*HIGH* high risk of bias, *LOW* low risk of bias, *N* no, *NA* not applicable, *NI* no information, *PN* probably no, *PY* probably yes, *RCTs* randomized controlled trials, *RoB* risk of bias, *SOME* some concerns, *Y* yes.

### DPT versus placebo on TMJ pain intensity at 12 weeks

**Figure 2:**
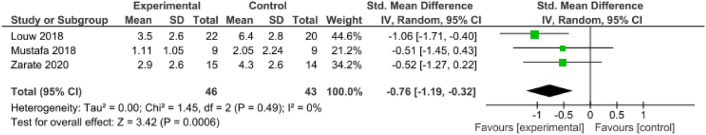
Dextrose versus Placebo injections for temporomandibular joint pain at 12 weeks.

In this comparison, three RCTs (n = 89) were eligible for pooling^21,22,28^. Visual Analog Scale (VAS) and numerical rating scale (NRS) were reported, with SMDs calculated in the random effect meta-analyses. Pooled results favored the use of DPT in reducing TMJ pain, with SMD − 0.76 (95% CI − 1.19 to − 0.32, P = 0.0006) and of low heterogeneity (I^2^ = 0%) (Fig. 2).

### DPT versus placebo on TMJ dysfunction at 12 weeks

**Figure 3:**
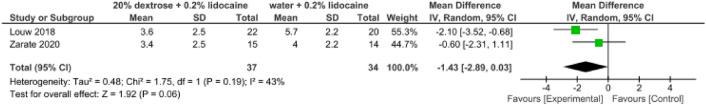
Dextrose versus Placebo injections for temporomandibular joint disability at 12 weeks.

Two RCTs (n = 71) were eligible for pooling; an NRS was used in both trials to assess TMJ dysfuction^21,22^. Although pooled results suggested a potential positive effect of DPT on reducing jaw disability, it was not statistically significant, with the weighted mean difference (WMD − 1.43; 95% CI − 2.89 to 0.03, P = 0.06, I^2^ = 43%) (Fig. 3).

### DPT versus placebo on MIO at 12 weeks

**Figure 4:**
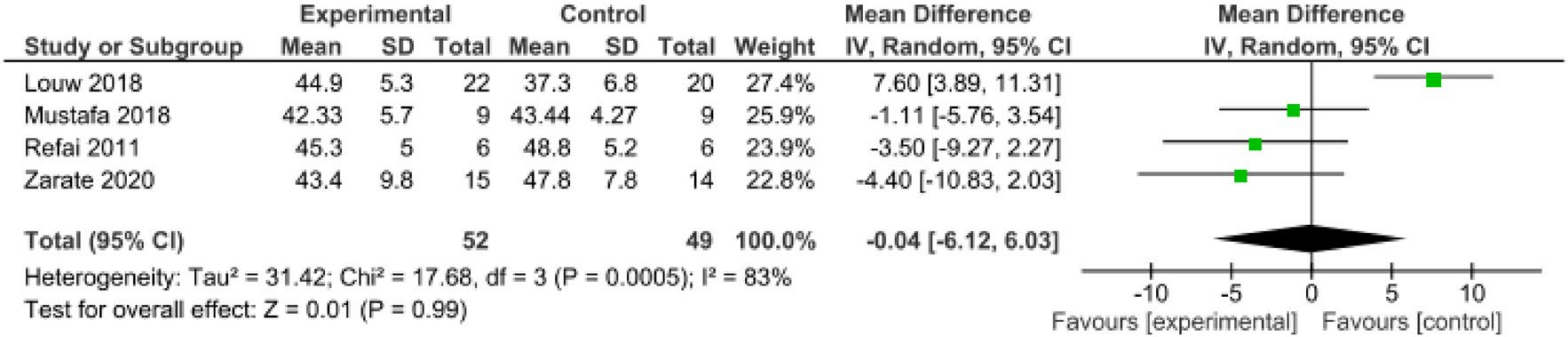
Dextrose versus Placebo injections for maximum incisor opening at 12 weeks.

Four RCTs (n = 101) were eligible for pooling^21,22,25,28^. In Refai et al. and Mustafa et al. (n = 30), combined intra-articular and extra-articular DPT injections suggested a trend of reducing MIO^25,28^. In Louw et al. and Zarate et al. (n = 71), only intra-articular injections were performed, with one favoring DPT and one favoring NS in reducing MIO^21,22^. Overall, the pooled data showed that there was no significant difference in the overall MIO between the DPT and placebo groups (md =  − 0.04, 95% CI − 6.12 to 6.03, I^2^ = 83%) (Fig. 4).

### DPT versus other active interventions

Pooling of results was not possible due to the use of different control interventions, different assessment time-points, and absence of raw figures in the publications. In Mahmoud et al., the use of platelet rich plasma demonstrated a statistically significant reduction in MIO compared to DPT and hyaluronic acid at 12 weeks, though no between-group differences were detected for pain scores^29^. In Hassanein et al., the use of laser therapy also resulted in a statistically significant reduction in MIO compared to DPT at 4 weeks; similarly, there were no between-group differences for pain scores^32^. In Arafet et al., the use of autologous blood was superior to DPT in reducing MIO at 2 and 4 weeks (P < 0.001), though longer term data was lacking^31^.

### Effectiveness of DPT at 12 months

In Kilic et al., no statistically significant improvement was observed between DPT and placebo groups at 12 months^26^. In Louw et al. and Zarate et al., DPT was offered to the control groups after participants were un-blinded at 12 weeks. The intra-group improvement in pain and function scores was sustained at 1 year, and inter-group difference was statistically significant in Louw et al. study, suggestive of longer term effectiveness^21,22^. However, the un-blinding and subsequent injection of DPT upon participant request, prevented us from including 12-month outcomes data in our meta-analysis.

### Adverse events

Adverse event-related outcomes were reported in 3 of the 10 included trials. One trial reported painful and burning sensations among 18 participants, with temporary paralysis of temporal branch of the facial nerve in 4 participants^27^. One trial reported one participant had worsening of jaw pain and swelling 2 months after study enrolment, and was subsequently diagnosed with an actinic cell tumor of the parotid gland unrelated to therapy^21^. One trial reported no adverse event reported throughout the study period^22^.

## Discussion

This study showed that DPT is superior to placebo injections in reducing TMJ pain intensity, with a moderate to large effect size and low heterogeneity at 12 weeks^33,34^. Although the findings do not demonstrate a statistically significant improvement in the disability score of DPT compared to placebo injections, the positive trend suggests that even in the context of meta-analysis, the comparison may be underpowered and that a larger sample size may be able to detect a difference. Comparison with other injection therapies such as corticosteroids and hyaluronic acid was not possible due to the absence of effect sizes in relevant TMJ reviews^35,36^.

Because different injection approaches were used in the included studies, special attention is needed in the interpretation of MIO findings. The normal values of MIO have been reported as 51.00 mm for male and 46.3 mm for female^37^. In the four included RCTs, Refai et al. and Mustafa et al. used the standard protocol of DPT consisting of intra-articular and extra-articular (capsular) injections. Participants in these trials had painful subluxation or dislocation of the TMJ; therefore, reducing MIO was expected to improve the overall joint stability through a “whole” joint treatment^25,28^. The finding was consistent with other prospective case-series, when extra-articular injections were found to reduce jaw motion^38,39^. Conversely, participants in the other two trials had painful clicking TMJ, without subluxation or dislocation; in these studies the effect of intra-articular DPT injection on joint stability was less consistent. Louw el at., reported an increase in MIO in the DPT group; Zarate et al., reported an increase in MIO in both groups^21,22^. We suggest that extra-articular injections, with multiple needling and the tissue proliferative effects of dextrose, may have recruited the inflammatory cascades leading to capsular strengthening^20^. Previous rodent studies of medial collateral ligaments injected with dextrose have reported increased levels of inflammatory markers in healthy tissue and an increased cross-sectional area in strain-injured tissue^16,17^. In rabbit models, injection of DPT into the connective tissue in the carpal tunnel produced thickening of the collagen bundles when compared with saline controls^18,19^. Although, we have not detected a statistically significant effect size on MIO, it appears possible that different protocols may be optimal for different sets of symptoms and signs. This view is supported by Fouda et al., who suggested that the selection of the injection site is the most important part of treatment, and that hypermobility should be treated with injection into the outer capsule, whereas pain is best treated with injection into the joint space^27^.

The mechanism by which DPT may decrease musculoskeletal pain, including TMD pain, is not well understood. Recruitment of the inflammatory cascade noted above may contribute to pain control through indirect, downstream wound healing effects. In addition, several models have been proposed which feature the direct effect of dextrose on nerve and other tissues. First, dextrose (d-glucose) is a crucial nutrient for functioning of cartilage and is the precursor for synthesis of glycosaminoglycans, glycoproteins, and glycolipids^40^. A recent in vitro study by Wu et al. showed that dextrose upregulates expression of aggrecan in chondrocytic ATDC5 cells and downregulates microRNA-14103-3p (miT141-3p). The resulting high local concentration of aggrecan may provide a favourable osmotic environment for growth and function of cartilage^41^ Second, dextrose solution hyperpolarises nerves by opening their potassium channels, thereby decreasing signal transmission in nociceptive pain fibres^42^. Third, glucose solutions may work by blocking transient receptor potential vanilloid type 1 (TRPV 1), a membrane cation channel that allows influx of sodium and calcium. Sodium influx is thought to result in an action potential and nociception, whereas calcium results in the release of substance P and calcitonin gene-related peptide^43^. Hence, blocking the influx of both cations may theoretically minimise neuropathic pain^44^. This mechanisms is consistent with recent preclinical and clinical data which strongly support a role for various TRP channels^45^. Clinically, a potential sensorineural analgesic mechanism of dextrose is suggested by its apparent effects in several clinical studies, including epidural injection of dextrose in the treatment of chronic low back pain^46^, intra-articular DPT injections for knee pain^47^, and significant pain reduction after perineural injection of DPT in patients with carpal tunnel syndrome or Achilles tendinitis^48,49^.

Strengths of the current study included timely conduct of study to review an area that is rapidly emerging, clinically important, and has disparate findings. Besides, we used rigorous methodology that conforms to best practice guidelines. There are several limitations in the current study. First, the number of included studies and total participant sample size were small. Second, raw data were missing in some articles as they were reported by plots and histograms; therefore, not all the data could be synthesized^29–31^. Third, changes in the diagnostic criteria of TMD resulted in a lack of diagnostic specificity across RDC/TMD categories in some studies, and some trials recruited participants with TMJ pain and others with hypermobility or subluxations. It is likely that patients in different diagnostic categories respond optimally to different injection protocols. Finally, the 12-week time frame available for data pooling was short. Therefore, longer term effects remain uncertain.

## Conclusion

In this systematic review and meta-analysis, evaluation of best available evidence shows that DPT conferred a large positive effect which met criteria for clinical relevance in the treatment of TMJ pain, compared with placebo injections. Therefore, in carefully selected patients, especially those with functional derangement of the TMJs and who are refractory to more conventional care, DPT can be considered an appropriate non-surgical treatment option. Selection of specific injection sites may best be informed by the presenting symptoms. Future rigorous research should include studies of longer-term follow-up. Direct comparison with other injection therapies, cost-effective analysis and a better understanding of mechanism of action will further inform the role of DPT in TMDs.

## Methods

We followed the statement on the Preferred Reporting Items for Systematic Reviews and Meta-Analyses for RCTs^50^. The protocol has been registered in the PROSPERO registry (CRD42020214305).

### Eligibility criteria

This review included parallel or cross-over RCTs that assessed the efficacy or effectiveness of DPT regardless of blinding or type of reporting^51^. For cross-over RCTs, only data before the wash-out period was used^52^. We excluded complex interventions in which DPT was not a sole treatment. Dissertations and conference abstracts were included if they contained sufficient details^53^.

### Information sources

Potential studies were identified by searching electronic databases including CENTRAL, MEDLINE, EMBASE, Web of Science, PubMed, Dimensions, Global Health, NHS Health Technology Assessment, AMED and OVID nursing database. The search period extended from their inception until 15th October 2020 and with no limitations on languages. The reference lists of the identified studies and relevant reviews on the subject were also scanned for additional possible studies.

### Search strategy

The search strategy was according to PICO design (Population, Intervention, Comparison, and Outcome). Keywords for population were: TMJ [all fields] OR temporomandibular joint [MeSH] OR dislocation [MeSH] OR joint hypermobility (MeSH) OR subluxation [all fields]; for intervention were: dextrose [MeSH] OR prolotherapy [MeSH] dextrose prolotherapy [all fields]; for comparison were: saline solution [MeSH] OR placebo effect [MeSH]; for outcomes were pain [MeSH] OR mouth opening [all fields] OR subluxation [all fields]. Search keys were summarized in Supplementary Appendix 1.

### Types of participants

This study included participants with TMD diagnosed by any pre-defined or specified diagnostic criteria, which fulfilled the Diagnostic Criteria/TMD Axis 1 (physical symptoms), regardless of age, race and gender^6^. Our study excluded patients with TMDs found to be caused by psychogenic or autoimmune inflammatory causes, which multidisciplinary care had been the core disease management^54,55^.

### Types of interventions

For inclusion, DPT had to be administered to at least one group within the trial. Consistent with the clinical practice of DPT, at least part of the injection protocol had to include an intra-articular injection, with or without additional injections to the peri-articular soft tissues.

### Types of comparison controls

Comparison groups could include saline, free water, any kind of active injections or interventions, or exercise. Co-interventions were allowed as long as they were uniform across all groups such that the net effect of DPT could be estimated.

### Outcome measures

The primary outcome of interest was pain intensity or pain relief in TMJ, measured by visual analogue scale (VAS), numerical rating scale (NRS), or algometry. Secondary outcomes included functional score, maximum inter-incisal mouth opening (MIO), frequency of locking or luxation, and number of adverse events.

### Study selection and data extraction

Two reviewers (RWWS, KDR) independently screened electronic retrieved titles and abstracts, evaluated potential relevant full texts and determined study eligibility. Copies of all articles of RCTs were obtained and read in full, and data from the articles were validated and extracted according to pre-defined criteria^56^. For eligible studies, data were extracted independently using a piloted data extraction form. For each eligible study, the following data were extracted: study design, participant characteristics, features of interventions, outcomes, duration of follow up and adverse events. An attempt was made to contact study authors regarding these methodological elements if not reported. Discrepancies in study selection and data extraction were resolved by third reviewer (DR).

### Risk of bias assessment

The Cochrane risk of bias (RoB) assessment tool 2 was used to evaluate the following 5 RoB domains: bias arising from randomization process; deviation from intended interventions; missing outcome data; measurement of outcome and selection of the reported results^57^. The RoB was assessed by two independent reviewers (CHLW, RWWS); any discrepancy was resolved by a 3rd reviewer (VCHC).

### Statistical analysis

All meta-analyses were conducted using the using Revman version 5.3^58^. A random effect model was used to pool study results, taking into account possible variations in effect sizes across trials^59^. Changes in continuous outcomes were pooled as standardized mean differences (SMD), with 95% confidence intervals (CI). Magnitude of the SMD was determined using standard approach: small, SMD = 0.2; medium, SMD = 0.5; and large, SMD = 0.8^33^. Weighted mean difference (WMD) was used to measure outcomes sharing the same unit of measure, and its potential clinical impact was interpreted according to the minimal clinical important difference (MCIDs) for TMD^60^. The I square (I^2^) statistic was calculated to estimate heterogeneity across studies. An I^2^ level of less than < 25%, 25–50% and greater than 50% were regarded as indicators of low, moderate and high levels of heterogeneity respectively^34^.

## Supplementary Information

Supplementary Information.
