# Supplementary material for: Efficacy of hypertonic dextrose injection (prolotherapy) in temporomandibular joint dysfunction: a systematic review and meta-analysis
Source: Sci Rep. 2021 Jul 19;11:14638. doi: 10.1038/s41598-021-94119-2 (PMC8289855; doi:10.1038/s41598-021-94119-2)
Supplement: Supplementary file 1 — Supplementary Information. [file 41598_2021_94119_MOESM1_ESM.pdf]

1. **MEDLINE via EBSCOhost**

Last searched September 16, 2020

S1. (MM "Temporomandibular Joint Disc") OR (MM "Temporomandibular Joint+") OR (MM "Temporomandibular Joint Disorders+") OR (MM "Temporomandibular Joint Dysfunction Syndrome") OR (MM "Arthrocentesis") OR "temporomandibular"

S2. TX TMJ

S3. TX TMD

S4. TX dextrose

S5. (MM "Prolotherapy") OR "Prolotherapy"

S6. (MM "Saline Solution") OR (MM "Saline Waters") OR (MM "Saline Solution, Hypertonic")

S7. TX water

S8. (MM "Lidocaine+") OR "lidocaine"

S9. TX xylocaine

S10. TX local anesthetic

S11. (MM "Exercise+") OR (MM "Plyometric Exercise") OR (MM "Exercise Test+") OR (MM "Exercise Therapy+") OR (MM "Exercise Movement Techniques+") OR (MM "Sports Nutritional Physiological Phenomena") OR (MM "Resistance Training") OR (MM "Sports Nutritional Sciences")

S12. TX usual care

S13. TX control group

S14. TX others

S15. S1 OR S2 OR S3

S16. S4 OR S5

S17. S6 OR S7 OR S8 OR S9 OR S10 OR S11 OR S12 OR S13 OR S14

S18. S15 AND S16 AND S17

2. **EMBASE, Global Health, AMED and Ovid Nursing Database via Ovid**

Last searched September 16, 2020

1. temporomandibular disorder*.tw.

2. temporomandibular dysfunction.tw.

3. exp *Temporomandibular Joint Disorders/ or exp *Temporomandibular Joint/ or temporomandibular.mp.

4. TMJ.tw.

5. TMD.tw.

6. Temporomandibular.tw.

7. dextrose.tw.

8. Prolotherapy.tw.

9. normal saline.tw.

10. water.tw.

11. lidocaine.tw.

12. xylocaine.tw.

13. local anesthetic.tw.

14. exercise.tw.

15. usual care.tw.

16. control group.tw.

17. others.tw.

18. 1 or 2 or 3 or 4 or 5 or 6

19. 7 or 8

20. 9 or 10 or 11 or 12 or 13 or 14 or 15 or 16 or 17

21. 18 and 19 and 20

3. CENTRAL

Last searched September 16, 2020

#1 MeSH descriptor: [Temporomandibular Joint Disorders] explode all trees

#2 temporomandibular dysfunction

#3 TMJ

#4 TMD

#5 Temporomandibular

#6 dextrose

#7 MeSH descriptor: [Prolotherapy] explode all trees

#8 MeSH descriptor: [Saline Solution] explode all trees

#9 normal saline

#10 water

#11 MeSH descriptor: [Anesthetics, Local] explode all trees

#12 MeSH descriptor: [Lidocaine] explode all trees

#13 xylocaine

#14 MeSH descriptor: [Exercise] explode all trees

#15 usual care

#16 control group

#17 others

#18 #1 OR #2 OR #3 OR #4 OR #5

#19 #6 OR #7

#20 #8 OR #9 OR #10 OR #11 OR #12 OR #13 OR #14 OR #15 OR #16 OR #17

#21 #18 AND #19 AND #20

4. **Web of science**

Last searched September 16, 2020

1 ALL=temporomandibular*

2 ALL=TMJ

3 ALL=dextrose

4 ALL=Prolotherapy

5 ALL=normal saline

6 ALL=water

7 ALL=lidocaine

8 ALL=xylocaine

9 ALL=local anesthetic

10 ALL=exercise

11 ALL=usual care

12 ALL=control group

13 ALL=others

14 #1 OR #2

15 #3 OR #4

16 #5 OR #6 OR #7 OR #8 OR #9 OR #10 OR #11 OR #12 OR #13

17 #14 AND #15 AND #16

5. **Pubmed**

Last searched September 16, 2020

((temporomandibular*) AND (dextrose OR Prolotherapy)) AND (normal saline OR water OR lidocaine OR xylocaine OR local anesthetic OR exercise OR usual care OR control group OR others)

6. **Dimensions**

Last searched October 4, 2020 (Limiters-Title and abstract)

(dextrose prolotherapy OR dextrose injection OR intra-articular OR prolotherapy) AND (temporo* OR temporomandibular joint OR temporo-mandibular joint) AND (dysfunction OR arthropathy OR pain OR disorder OR hypernociception OR hypermobility)

7. **NHS Health Technology Assessment**

Last searched September 16, 2020

#1 MeSH descriptor: [Temporomandibular Joint Disorders] explode all trees

#2 temporomandibular dysfunction

#3 TMJ

#4 TMD

#5 Temporomandibular

#6 dextrose

#7 Prolotherapy

#8 MeSH descriptor: [Saline Solution, Hypertonic] explode all trees

#9 normal saline

#10 water

#11 MeSH descriptor: [Anesthetics, Local] explode all trees

#12 MeSH descriptor: [Lidocaine] explode all trees

#13 xylocaine

#14 MeSH descriptor: [Exercise] explode all trees

#15 usual care

#16 control group

#17 others

18 #1 OR #2 OR #3 OR #4 OR #5

19 #6 OR #7

20 #8 OR #9 OR #10 OR #11 OR #12 OR #13 OR #14 OR #15 OR #16 OR #17

21 #18 AND #19 AND #20
